# An Important Matter

**Published:** 1868-12-15

**Authors:** 


### *An Important Matter.*

The position of Medical Inspector during the recent war was found one of great importance both to the army and the government. The former were better provided for, and, meanwhile, the interests of the latter were subserved.

In common with our fellow-citizens, we desire to have all unnecessary governmental expenses put an end to; but we yet believe in the scriptural statement: "There is that scattereth, and yet increaseth — there is that withholdeth more than is meet, and cometh to poverty." A business man who is under the pressure of large liabilities would scarcely be credited with prudence, should he dispense with his bookkeeper and necessary overseers of the work; and so the government can not afford to dispense with the important office of Medical Inspector of Marine Hospitals, etc. Such an officer, if properly chosen, will save to the people ten times his salary in a single year. Aside from his assisting in the better development of the hospitals in a sanitary point of view, he can prove most useful in stopping pecuniary leakages. One notable instance of this latter service has come to our own immediate knowledge. A faithful public servant of this sort saved a large amount to the government by careful inspection of a single hospital. The Journal chronicles his name with peculiar pleasure. We refer to the accomplished Inspector and Special Agent, W. D. Stewart, M.D., whose recent visit to Western hospitals was noticed in a former number of the Journal. A fine professional scholar, of large practical experience and unswerving integrity, he is exactly the right man in the right place, and we trust that amid whatever official changes may be made, he will be retained, both for the credit of the profession and the advantage of the public. Of his politics we know nothing, and we do not scruple to say we care less. *Fiat justitia.*

